# Supplementary material for: Global hypo-methylation in a proportion of glioblastoma enriched for an astrocytic signature is associated with increased invasion and altered immune landscape
Source: eLife. 2022 Nov 22;11:e77335. doi: 10.7554/eLife.77335 (PMC9681209; doi:10.7554/eLife.77335)
Supplement: Figure 2—source data 1. [file elife-77335-fig2-data1.zip › Figure_2_source_data_1/Figure_2B/homerResults/motif35.similar.html]

motif35

## Information for motif35

G
A
C
T
C
A
G
T
T
A
C
G
C
T
G
A
T
A
C
G
A
T
G
C
G
T
A
C
A
T
C
G
  
Reverse Opposite:  

T
A
G
C
C
A
T
G
T
A
C
G
A
T
G
C
G
A
C
T
A
T
G
C
G
T
C
A
C
T
G
A
  

|  |  |
| --- | --- |
| p-value: | 1e-42 |
| log p-value: | -9.697e+01 |
| Information Content per bp: | 1.486 |
| Number of Target Sequences with motif | 88.0 |
| Percentage of Target Sequences with motif | 3.81% |
| Number of Background Sequences with motif | 2.6 |
| Percentage of Background Sequences with motif | 0.73% |
| Average Position of motif in Targets | 97.7 +/- 56.9bp |
| Average Position of motif in Background | 189.0 +/- 0.0bp |
| Strand Bias (log2 ratio + to - strand density) | 0.3 |
| Multiplicity (# of sites on avg that occur together) | 1.04 |
| Motif File: | file (matrix) reverse opposite |

### Similar de novo motifs found

|  |  |  |  |  |  |  |  |
| --- | --- | --- | --- | --- | --- | --- | --- |
| Rank | Match Score | Redundant Motif | P-value | log P-value | % of Targets | % of Background | Motif file |
| 1 | 0.607 | A G T C A C T G A C T G A C T G A C G T C G T A C G T A C G T A | 1e-25 | -58.821633 | 5.24% | 1.78% | motif file (matrix) |
| 2 | 0.626 | A G T C A C T G A C T G A G T C A C G T C G T A A G T C C G T A | 1e-24 | -56.964132 | 5.67% | 2.16% | motif file (matrix) |
| 3 | 0.644 | A C G T A C T G A C G T A C G T A G T C C G T A | 1e-24 | -56.074417 | 15.79% | 9.06% | motif file (matrix) |
